# Ecological Porous Concrete: A Review of Multi-Scale Pore Structure Engineering for Coupled Mechanical and Ecological Performance

**DOI:** 10.3390/ma19132873

**Published:** 2026-07-05

**Authors:** Wenjing Zhao, Yalin Li, Linan Gu, Fangzhou Ren, Miao Miao, Jingjing Feng

**Affiliations:** 1 College of Mechanical and Electronic Engineering, Shandong Agricultural University, Taian 271018, China; 2024010166@sdau.edu.cn; 2 College of Water Conservancy and Civil Engineering, Shandong Agricultural University, Taian 271018, China; gu_linan@sdau.edu.cn (L.G.); 1113300419@hit.edu.cn (F.R.); miaom@sdau.edu.cn (M.M.); 3 Jiangsu Key Laboratory of Mechanical Analysis for Infrastructure and Advanced Equipment, Department of Engineering Mechanics, School of Civil Engineering, Southeast University, Nanjing 210096, China; 230238349@seu.edu.cn

**Keywords:** ecological porous concrete, multi-scale pore structure, interfacial transition zone, alkalinity regulation, plant compatibility, cross-scale synergy

## Abstract

**Highlights:**

- The porosity–strength trade-off is analyzed across micro-, meso-, and macro-scales.
- A micro-strengthening, meso-facilitation, and macro-moderation strategy is proposed.
- Key control points are ITZ, pore throat geometry, and total porosity.

**Abstract:**

Ecological porous concrete (EPC) offers both structural performance and ecosystem services, yet an inherent contradiction exists between the ecological benefits of high porosity and mechanical performance. Traditional design methods focusing solely on macro-scale porosity fail to achieve synergistic optimization. This review comprehensively synthesizes the intrinsic correlations between EPC’s multi-scale pore structures and key properties from micro-, meso-, and macro-scale perspectives, drawing upon representative studies across experimental, numerical, and theoretical approaches. The microscale reveals interfacial transition zone bonding, capillary pore effects, and alkalinity regulation for vegetation compatibility. The mesoscale clarifies the control of effective porosity, tortuosity, and pore throats on fluid transport and root penetration. The macro-scale analyzes skeletal pore support for plant growth, hydrology, and slope stability. A cross-scale collaborative design approach is proposed, featuring microscopic reinforcement, mesoscopic continuity, and macroscopic moderation. This paper provides theoretical support for EPC’s transition from empirical to precision design, promoting low-carbon and large-scale applications in revetments, Sponge Cities, and slope restoration.

## 1. Introduction

Under the dual drivers of Sponge City construction and ecological civilization development, the development of building materials that integrate structural strength with ecosystem services has become a core issue in sustainable engineering [1,2]. As an environmentally friendly composite material, ecological porous concrete (EPC) has gradually evolved into an ecological engineering solution integrating multiple functions such as water purification [3,4], soil and water conservation, and vegetation restoration [5,6,7]. Material performance in service is fundamentally determined by its complex multi-scale pore system, comprising nanoscale and microscale capillary pores within the cement paste, millimeter-scale inter-aggregate voids, and centimeter-scale layered structures [8,9,10]. This structural system spans multiple spatial dimensions, with pore characteristics at different scales governing key properties such as mechanical evolution, moisture transport, and plant compatibility [11,12].

**Figure 1:**
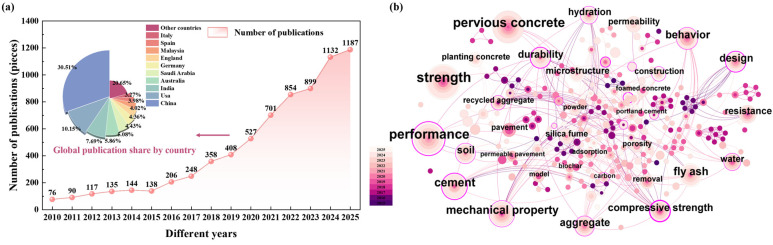
Bibliometric analysis and knowledge mapping of EPC research: (**a**) annual publication trends and country distribution (2010–2025); (**b**) keyword co-occurrence clustering network (2015–2025). Bibliometric data were retrieved from the Web of Science Core Collection covering publications from 2010 to 2025. The search strategy was: TS = (“porous vegetation” AND “concrete”) OR “porous concrete” OR (“Vegetation” AND “concrete”) OR “Plant-Growing Concrete” OR (“ecological” AND “concrete”) OR “Green concrete” OR (“environmentally*” AND “concrete”). Document types were limited to articles and reviews. Duplicate records were removed prior to analysis. A total of 7220 publications were retrieved from the database for the bibliometric analysis.

Keyword clustering and bibliometric analysis reveal a significant shift in the research focus of ecological concrete (Figure 1). Early studies primarily concentrated on mix proportion design and empirical correlations between basic mechanical properties and water permeability coefficients. Current research has pivoted toward multifunctional integration, long-life durability design, and low-carbon production technologies [4]. Quantitative assessments indicate that the carbon footprint of EPC can be substantially reduced through various strategies. Replacing 50% of the cement with ground granulated blast furnace slag reduces carbon emissions by up to 45% [13]. The combination of recycled aggregate with low-carbon blended cements can also provide reductions of up to 25% [14]. Life-cycle assessments further reveal that EPC can achieve a net carbon sink of 351–397 kg CO_2_eq over a 50-year service life [15]. In slope protection applications, it cuts total carbon emissions by 667.21 tons compared to conventional concrete, is projected to achieve carbon neutrality within 3.66 years, and delivers a net carbon sequestration benefit of 2422.97 tons over its lifecycle [16]. Particularly under policy drivers such as Sponge City and ecological restoration, balancing the trade-off in mechanical strength with durability degradation caused by high porosity has become the core driver of multi-scale pore structure studies [17]. Research in China, the United States, and Australia on high-performance ecological concrete modification and multi-field coupled degradation mechanisms is leading the field toward precision design [18].

Nevertheless, current studies primarily rely on total porosity (*P*) as the key characterization parameter, often overlooking the fundamental contradiction between high porosity (ecological performance) and high density (structural performance) [19,20]. In recent years, significant progress has been made in EPC research concerning mix design, mechanical-permeability relationships, plant compatibility, and durability [21]. Several reviews have addressed pervious or ecological concrete from specific perspectives. Some studies have summarized the empirical relationships among mix design, mechanical properties, and permeability [22]. Others have focused on the plant compatibility and durability of vegetated pervious concrete [8,23]. Still others have discussed the effects of aggregate type and admixtures on performance from a materials preparation perspective [24]. However, the cross-scale linkages among microscopic mechanisms, mesoscopic characteristics, and macro-scale performance remain unclear, and how interactions across scales influence the service behavior of EPC is still poorly understood. This review therefore examines the progress in EPC across three scales. The microscale covers interfacial transition zone (ITZ) formation and regulation, alkalinity management, and pore evolution. The mesoscale addresses connectivity, tortuosity (*τ*), and pore throat characteristics. The macro-scale involves plant growth, hydrological regulation, and engineering performance. The aim is to reveal the cross-scale correlation mechanisms from pore formation to service performance and to outline design criteria for engineering applications, providing theoretical support for the transition of EPC from empirical mix design to performance-oriented precision design.

It should be noted that as a narrative review, the references cited in this paper are not an exhaustive inclusion of all 7220 retrieved publications. We adopted the following selection strategy: priority was given to recent publications after 2020, with particular emphasis on studies published after 2023, to ensure that the latest advances in the field are adequately reflected. At the same time, influential classical literature was also retained to ensure the completeness of the fundamental theoretical foundation. Within this framework, three principles were followed: (1) prioritizing representative works (both classic contributions and recent breakthroughs) in each research direction; (2) selecting typical studies that support the discussions at the micro-, meso-, and macro-scales, with multi-scale pore structure as the guiding thread; and (3) covering different research approaches including experimental studies, numerical simulations, and theoretical analyses. The cited references span a range of topics, including material composition, mix design, microstructural characterization, mechanical properties, permeability, plant compatibility, durability, and engineering applications, offering a useful overview of the current state of EPC research.

## 2. Overview of EPC Technology

### 2.1. Definition and Classification of EPC

**Figure 2:**
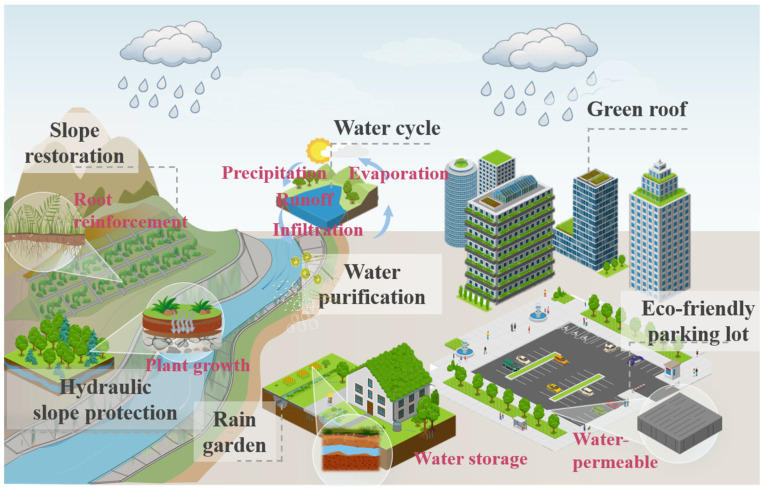
EPC typical application scenarios illustration and ecological functions.

EPC represents a key category of green building materials. It is a composite material fabricated through gradation optimization and pore structure regulation, integrating structural performance suitable for low-load applications with ecosystem services [25]. By incorporating plants and soil media into the concrete matrix, it realizes the symbiotic integration of vegetation and infrastructure. As shown in Figure 2, EPC is widely applied in fields including slope protection, urban greening, and ecological parking lots, forming a comprehensive ecological engineering system ranging from aquatic environments to building facades.

The current academic nomenclature for porous ecological materials is function-oriented. From a structural perspective, porous concrete predominantly denotes interconnected pore features [26,27]. Porous Concrete refers to permeable properties [28,29] and ecological concrete encompasses environmental functions [30,31]. However, when addressing vegetation integration, Porous Concrete alone is insufficient. Terms such as Porous Vegetation Eco-concrete and Ecological Porous Concrete share the same denotation as Vegetated Porous Concrete. In subsequent sections, EPC will be used uniformly to refer to this category.

### 2.2. Structural Characteristics of EPC

In typical EPC structures, the bottom layer comprises porous concrete to protect the stability of the underlying soil, while the upper layer consists of soil containing fertilizers, water-retaining agents, and plant seeds to support initial vegetation growth [4]. Over time, the excellent permeability and aeration provided by the porous structure [32] facilitate soil infiltration into concrete pores, enabling plant roots to establish a stable network that significantly enhances soil anti-scouring capacity and further stabilizes the underlying substrate [33].

**Figure 3:**
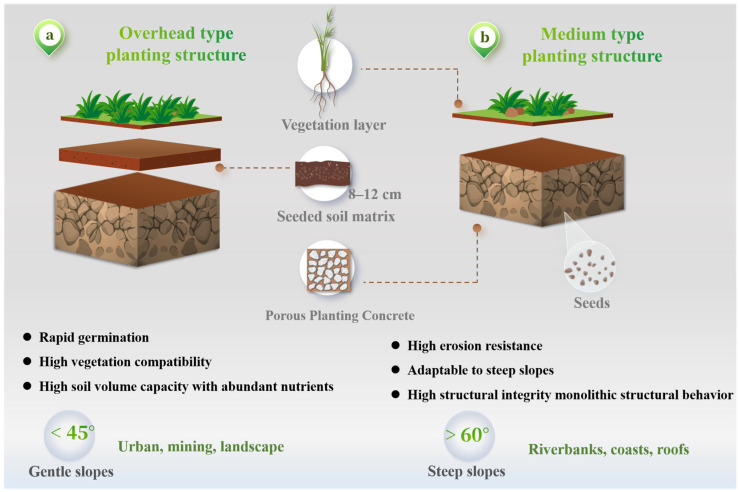
Two typical structural configurations and application scenarios of EPC (**a**) Overhead type planting structure; (**b**) Medium type planting structure.

According to the combination method of soil layer and concrete layer, EPC is mainly divided into two typical structural forms (Figure 3). Among them, the overhead type physically separates the concrete layer from the soil layer, achieving drainage and ventilation through an elevated space [34]. This structure offers advantages such as rapid turf formation, wide plant adaptability, and large soil capacity, making it suitable for gentle slopes with gradients below 45°, such as municipal greening, mine restoration, and landscape projects. The Medium type fills soil into the pores of the concrete to form a composite whole. It exhibits strong anti-scouring capability, adaptability to steep slopes exceeding 60°, lightweight structure, and good integrity, commonly used in projects like riverbank protection, coastal defense, and rooftop greening [35].

## 3. Microscale Pore Structure

Porosity, defined as the percentage of pore volume to the total volume of a material, is the most direct and primary parameter affecting concrete strength. For EPC, macropores (large inter-aggregate voids, 15–30%) are significantly more prevalent than in conventional concrete, ensuring permeability and vegetation functions but resulting in lower compressive strength (7–25 MPa) [36]. The presence of pores reduces the effective load-bearing cross-sectional area of the material and acts as stress concentration points under load, triggering the initiation and propagation of microcracks [37,38]. At the microscale characteristics (<10 μm capillary pores and ITZ structures), while not directly determining strength values, these features govern interfacial bonding quality, durability degradation pathways, and the chemical environment for plant growth. The ITZ determines the bonding stability between aggregates, the distribution of micropores governs water retention capacity and ion transport rate, and the alkalinity of pore solution dictates plant compatibility. Therefore, the microstructure serves as the key to understanding the strength and eco-synergistic mechanisms of ecological concrete.

### 3.1. Formation Mechanism and Failure Modes of ITZ

EPC typically employs a semi-dry stiff paste with low water-to-binder ratio (the commonly used ratio is 0.35–0.45), which results in distinctive microstructural characteristics. The paste content is relatively low, and its hydration process is considerably influenced by the pore environment. Consequently, its mechanical properties are highly dependent on the compactness of the paste at aggregate bonding interfaces [39]. The ITZ in EPC has a physical thickness typically ranging from 20 to 50 μm, which places it in the mesoscale range by dimensional classification. However, as the primary site of crack initiation under compressive loading, its interfacial bonding quality and chemical environment (e.g., Ca(OH)_2_ concentration and alkalinity) are governed by microscale mechanisms [40]. Therefore, in this review, the ITZ is discussed within the micro-scale chapter to emphasize its role as the origin of mechanical damage and chemical interaction, a dual treatment that reflects its cross-scale nature, while acknowledging that its physical dimensions belong to the mesoscale [41]. The ITZ, with its low paste content and poor wettability, exhibits a loose structure that becomes a weak mechanical link. In these highly porous concretes, the problem is further amplified. Stress becomes highly concentrated under load, while the shrinkage stress at the edges of large pores induces microcracks in the ITZ during the early hardening stage. The lack of fine aggregate cushioning leads to rapid crack propagation.

**Figure 4:**
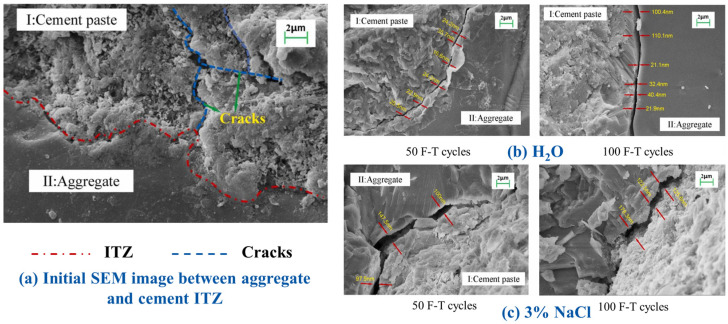
SEM micrographs of aggregate-cement ITZ in EPC: (**a**) initial state without F-T cycles; (**b**) specimens subjected to 50 and 100 F-T cycles under pure water environment; (**c**) specimens subjected to 50 and 100 F-T cycles under 3% NaCl solution environment [42].

**Figure 5:**
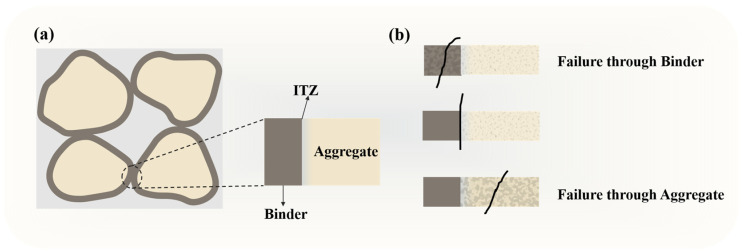
Schematic illustration of the (**a**) ITZ structure and (**b**) three typical failure modes in EPC.

As shown in Figure 4, interfacial degradation progressed with multiple freeze–thaw (F-T) cycles, leading to macroscopic strength loss [42]. Recycled and coral aggregates have seen increasing use. These materials not only exacerbate paste deficiency in the interfacial zone but may also fracture internally under load. This transforms the traditional ITZ into a composite weak zone encompassing both the aggregates and the paste [43,44]. Therefore, the failure modes of EPC can be summarized as the fracture of the bonding layer between aggregate particles, fracture at the aggregate–matrix interface, and aggregate fracture [37], as illustrated in Figure 5. However, it should be noted that these modes are not mutually exclusive, and mixed-mode failure often dominates under realistic loading [45]. Furthermore, although recycled aggregates are widely reported to exacerbate ITZ weakness, recent evidence suggests that their inherent porosity can buffer stress concentration and delay crack coalescence when paste encapsulation remains intact. Thus, old mortar may act as a compliant interlayer that mitigates stress concentration and reduces the crack driving force [46].

### 3.2. Microscale Pores: Strength Effects and Water Retention Mechanisms

Although EPC is characterized by macroscopic pores, the microscopic pores within the paste determine its mechanical strength. C–S–H gel pores smaller than 100 nm constitute part of the solid skeleton and have a negligible effect on strength. Capillary pores ranging from 10 nm to 10 μm, especially those larger than 1 μm, act as stress concentrators and significantly weaken the matrix strength. Therefore, reducing harmful capillary pores is crucial for strength enhancement [47]. On the other hand, these abundant micro- and nano-scale capillary pore networks function as powerful Micro-Reservoirs that adsorb and retain substantial water via capillary action. This water retention capacity provides continuous moisture supply to plant roots during non-rainfall periods, serving as a critical microscopic mechanism enabling EPC to achieve the hydrological function of rapid drainage and long-term water retention [48,49].

Besides pore size, the heterogeneity of pore size distribution also influences compressive strength. A more dispersed pore size distribution, characterized by a mixture of pore sizes, typically results in lower compressive strength [50], as large pores dominate failure while small pores cannot compensate for the resulting strength loss. Geometrically, the fractal dimension of pores shows a strong negative correlation with compressive strength; higher fractal dimension values indicate more complex and irregular pore structures, which cause greater strength deterioration [51].

### 3.3. Alkalinity Control and Alkali Reserve Mechanisms

**Figure 6:**
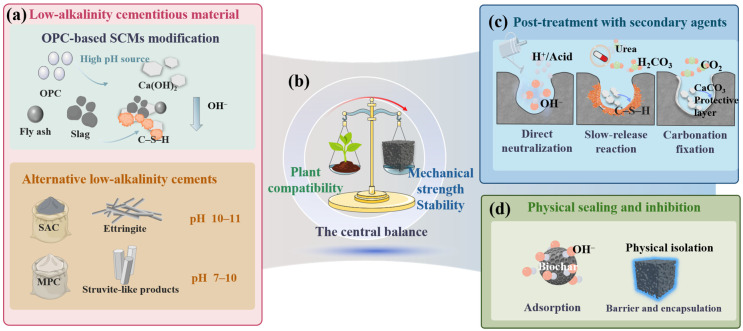
Classification and microscopic mechanisms of alkalinity regulation strategies in EPC: (**a**) low-alkalinity cementitious materials, (**b**) balance between plant compatibility and mechanical strength, (**c**) secondary alkali-reducing agents, (**d**) physical alkali sealing.

Portland cement (OPC) exhibits high alkalinity with pore solution pH typically exceeding 12 [52]. Excessively high alkalinity significantly inhibits plant growth, while insufficient alkalinity compromises the mechanical strength and structural stability of EPC [53]. Additionally, the accumulation of soluble salts (e.g., sodium carbonate, sodium bicarbonate) creates salt stress, slowing photosynthesis, restricting mineral nutrient uptake, and disrupting cellular osmotic balance [54]. Alkalinity regulation strategies are designed to achieve a balance between plant compatibility and mechanical strength (Figure 6b), which serves as the guiding principle for the approaches discussed below [55]. As shown in Figure 6, these strategies can be classified into three categories, corresponding to Section 3.3.1, Section 3.3.2 and Section 3.3.3: low-alkalinity cementitious material systems (Figure 6a) reduce pore solution pH at the source through OPC blended with supplementary cementitious materials (SCMs) or alternative low-alkalinity cements; secondary alkali-reducing agents (Figure 6c) achieve post-treatment alkalinity reduction through acid neutralization, slow-release reactions, and carbonation fixation; and physical alkali sealing (Figure 6d) relies on adsorption and surface barriers to physically isolate alkaline ion migration [9,56].

#### 3.3.1. Low-Alkalinity Cementitious Material Systems

Low-alkalinity cementitious material systems encompass diverse technical routes for reducing pore solution pH (Figure 6a) [57]. Among these, alkali-reducing supplementary cementitious materials SCMs represent the mainstream approach, referring to industrial by-products that consume alkaline substances through pozzolanic reactions with OPC hydration products [58]. Primarily composed of siliceous-aluminous glassy phases, these materials enable alkalinity reduction while facilitating solid waste valorization. The mechanism underlying alkalinity reduction by admixtures such as fly ash, silica fume, and slag involves the secondary hydration of their active components (SiO_2_, Al_2_O_3_) with Ca(OH)_2_, converting strong alkalis into low-alkalinity amorphous gels such as C–(A)–S–H, thereby reducing OH^−^ concentration in the pore solution [53]. Beyond SCMs, low-alkalinity binder systems include magnesium phosphate cement (MPC) and calcium sulfoaluminate cement (SAC) [59]. The pore solution pH of hydrated MPC ranges from 7 to 10; however, its long-term strength stability is poor, and ammonia gas released during hydration poses environmental hazards, limiting its application in ecological engineering [60]. The main hydration products of SAC are ettringite (AFt) and minor C–S–H [30], rendering it suitable for low-alkalinity concrete preparation [61]. Nevertheless, OPC remains widely used due to its cost-effectiveness, setting time controllability, and extensive engineering experience. However, the long-term effectiveness of these low-alkalinity systems remains debated. These inconsistencies arise from the divergent chemical stability of hydration products under service exposure. SAC achieves a low initial pH through AFt formation, yet AFt is metastable and susceptible to carbonation-induced decomposition, which raises pH rebound risks and undermines long-term microstructural integrity [62]. Low-calcium fly ash relies on slow pozzolanic consumption of Ca(OH)_2_; when the calcium content is insufficient or the reactivity is low, the alkalinity reduction is delayed and the strength development is retarded [63]. Therefore, the effectiveness of low-alkalinity systems depends not only on the initial pH but also on the carbonation resistance of the hydration product assemblage and the calcium availability for secondary reactions. Wu et al. demonstrated that multi-solid-waste-based low-alkalinity cementitious materials can achieve source alkalinity reduction with plant compatibility [55]. Their long-term performance, however, requires further validation. These inconsistencies suggest that alkalinity control cannot be adequately evaluated by single-point pH measurements. The interplay among binder chemistry, curing history, and environmental exposure should be considered collectively.

#### 3.3.2. Secondary Alkali-Reducing Agent

Unlike internal alkali-reducing SCMs, secondary alkali-reducing agents refer to technologies that regulate alkalinity through spraying, immersion, or curing after concrete casting (Figure 6c) [64]. Based on their mechanisms of action, they can be further classified into three types: direct neutralization, slow-release reaction, and carbonation fixation. Direct neutralization agents consume OH^−^ in the pore solution by introducing acidic substances. Acidic solutions such as citric acid, oxalic acid, and ferrous sulfate are the most commonly used secondary alkali-reducing agents [65]. Research by Zhuang et al. [66] demonstrated that adding 0.4% acetic acid reduced the pH by 0.65 units (from 10.3 to 9.65), while simultaneously improving compressive strength by 40.29% due to microstructural optimization. Zhu et al. [67] proposed using aluminum potassium sulfate to neutralize alkalinity, wherein hydrolysis of Al^3+^ generates H^+^ to consume OH^−^, regulating pH to a range suitable for plant growth (pH < 10.0). The mechanism of slow-release reactive alkali-reducing agents differs from direct neutralization. Taking urea as an example, Zhou et al. [68] demonstrated that the pH of EPC decreased from 11.0 (1 d) to 9.0 (28 d) during soaking, and the addition of 3.5 wt.% urea further reduced the 28 d pH by approximately 0.3 units compared to the control. The mechanism involves urea hydrolysis to generate carbonic acid, which then reacts with Ca(OH)_2_ from cement hydration while releasing water, thereby producing a dilution effect.

Carbonation-based alkali reduction utilizes the reaction between atmospheric CO_2_ and cement hydration products. The open-pore structure of vegetation concrete facilitates CO_2_ diffusion, allowing it to react with Ca(OH)_2_ to form neutral CaCO_3_ and create a protective layer on both the surface and interior. This slows down the release of OH^−^ and achieves gradual alkalinity reduction [69]. Compared to natural carbonation, accelerated carbonation curing is more effective, but prolonged treatment may cause strength loss [2]. Carbonation exhibits distinct short-term and long-term effects on EPC performance. In the short term, moderate carbonation reduces pore solution alkalinity, creating favorable conditions for seed germination and root establishment [70]. In the long term, however, progressive carbonation depletes Ca(OH)_2_ reserves and decalcifies C–S–H gel [69]. The resulting pore structure loosening reduces mechanical strength and accelerates subsequent degradation [71]. This risk is amplified for low-alkalinity cementitious systems such as SAC and MPC, whose hydration products exhibit inherently lower carbonation resistance compared to OPC-based systems [62]. It should also be noted that, unlike conventional exposed concrete, EPC in service is typically covered by soil and vegetation layers that significantly impede CO_2_ ingress, rendering natural carbonation a slow and surface-limited process.

Regarding the strength issues after alkali reduction treatment, current research findings remain inconsistent. Most reports indicate a compressive strength loss ranging from 10 to 30%. These seemingly contradictory outcomes can be attributed to differences in treatment protocols and material systems. When excessive acid is introduced or the liquid-to-solid ratio is poorly controlled, aggressive leaching of Ca(OH)_2_ and decalcification of C–S–H gel occur, directly degrading the cementitious matrix and causing measurable strength loss [65]. Conversely, under optimized dosage and controlled reaction conditions, mild acid neutralization or carbonation can refine pore structure by precipitating secondary calcite within capillary pores, thereby densifying the microstructure and marginally improving strength [72]. Furthermore, the baseline binder system matters: OPC-based EPC with abundant Ca(OH)_2_ reserves tolerates moderate alkali reduction better than low-calcium systems such as SAC, where hydration products are inherently less stable upon chemical alteration [62]. However, beyond these mechanistic considerations, the practical application of these secondary treatments faces challenges in dosage control, temporal effectiveness, and spatial uniformity. The optimal dosage range is often narrow, and either excess or deficiency compromises the desired pH reduction [1,65]. In terms of temporal effectiveness, the pH reduction achieved by direct neutralization tends to diminish over time as cement hydration continues [70]. Spatially, immersion treatments are more effective than surface spraying, as the latter exhibits a pronounced gradient effect with significant pH reduction concentrated in the near-surface layer [73,74].

#### 3.3.3. Physical Alkali Sealing

Physical alkali sealing inhibits the migration of alkaline ions into the pore solution through physical adsorption, dilution, or surface barrier effects, thereby avoiding the potential strength loss associated with chemical alkali reduction (Figure 6d). Adsorption- and dilution-based physical alkali sealing is primarily achieved by incorporating functional fillers. Porous admixtures such as biochar can adsorb alkaline ions in pore solutions, reducing alkali leaching [75]. High-volume mineral admixtures dilute cement content, thereby reducing alkali generation at the source. Barrier- and encapsulation-based physical alkali sealing isolates alkali migration by forming physical barriers on or within material surfaces. Paraffin wax is the most commonly used physical coating material [72]. Unlike chemical treatments, it does not damage the concrete structure and causes almost no loss of strength. Silane and epoxy resin are also frequently employed for surface treatment, forming dense barriers on material surfaces that function rapidly without reducing substrate strength [76,77]. Deep Penetration Sealer (DPS) is a water-based permeable crystalline coating. Research shows it effectively blocks alkali migration, reducing pH to 7.8 with subsequent stabilization, while slightly improving strength [2].

### 3.4. Microscale Deterioration Mechanisms and Control

The high porosity of EPC, while supporting ecological functions, also increases the risk of harmful ion intrusion and F-T damage. Chloride ions and sulfate ions are the primary harmful ions, with sulfate attack posing a more significant threat. Chloride ions chemically react with C_3_A (3CaO·Al_2_O_3_) in cement hydration products to form low-solubility Friedel’s salt, a process known as chemical fixation [78]. The incorporation of fly ash and ground granulated blast furnace slag can enhance this reaction, thereby mitigating chloride attack. Sulfate attack manifests as dual-mode chemical and physical deterioration: chemically, it reacts with Ca(OH)_2_ to form gypsum and AFt, with product volume expansion of approximately 1.4–2.2 times, generating micro internal stresses [79]. Physically, in tidal wet–dry cycles, sulfate crystallization creates pressure that damages pore structures.

F-T action accelerates microstructural deterioration, as the pore water freezing during F-T cycles generates internal stress and induces microcracks [80]. The reduction in compactness leads to an increase in pore structure parameters such as porosity, critical pore size, and most probable pore size [81]. Research by Luo et al. [82] demonstrated that after 30 F-T cycles, EPC exhibited a 15% reduction in flexural fatigue life and a 50% increase in fatigue failure rate, significantly impacting EPC in seasonally frozen regions.

In tidal zones, seasonally rainy regions, and areas with fluctuating groundwater levels, EPC is frequently subjected to alternating wetting and drying cycles. During the wetting phase, water and dissolved aggressive ions penetrate the pore network, while the drying phase induces salt crystallization and the concentration of harmful ions within the pores [83]. The resulting crystallization pressure and repeated volumetric changes progressively weaken the pore structure and degrade the aggregate–paste interface. Studies have shown that sulfate attack under wetting–drying cycles causes more significant performance loss than under continuous immersion, with compressive strength losses reaching up to 13.4% after 150 cycles [84]. Moreover, the deterioration caused by wetting–drying cycles follows a distinct mechanism from F-T damage: wetting–drying primarily induces dissolution and loss of pore wall materials in the early stage, while F-T cycles mainly cause crack initiation and propagation in the later stage [42,58].

### 3.5. Microstructure Optimization Approaches

To address the aforementioned microscopic issues, such as weak ITZ, alkalinity imbalance, and ion erosion, it is necessary to enhance the interfacial bonding and chemical environment stability by adjusting the paste microstructure while maintaining the macroporosity [36]. The main optimization approaches and their mechanisms are shown in Table 1.

## 4. Mesoscopic-Scale Pore Structure

The mmesoscale(10 μm–10 mm, comparable to coarse aggregate particle size) constitutes the core hierarchical level of vegetated concrete’s pore structure, corresponding to the skeletal pore network formed by cement paste bridges between aggregate particles. The porosity at this scale (typically 15–30%) not only determines the fluid permeability coefficient (on the order of 2–15 mm/s) and ion diffusion pathways, but more importantly, forms the physical accommodation space for root extension and serves as the retention carrier for matrix filling. As ITZ assemblies between aggregate and paste, meso-scale pores serve as structural weak zones with stress concentration factors reaching 3–5, governing the initiation, propagation, and cross-scale evolution of microcracks into macroscopic deterioration. Therefore, analyzing the topological characteristics of meso-pores (connectivity, *τ*, pore throat distribution) is the key to establishing a multi-performance synergistic mechanism for vegetated EPC structures with ecological and durable properties.

### 4.1. Connectivity Mechanism Between Aggregates: (P_e_) and Total Porosity (P)

**Figure 7:**
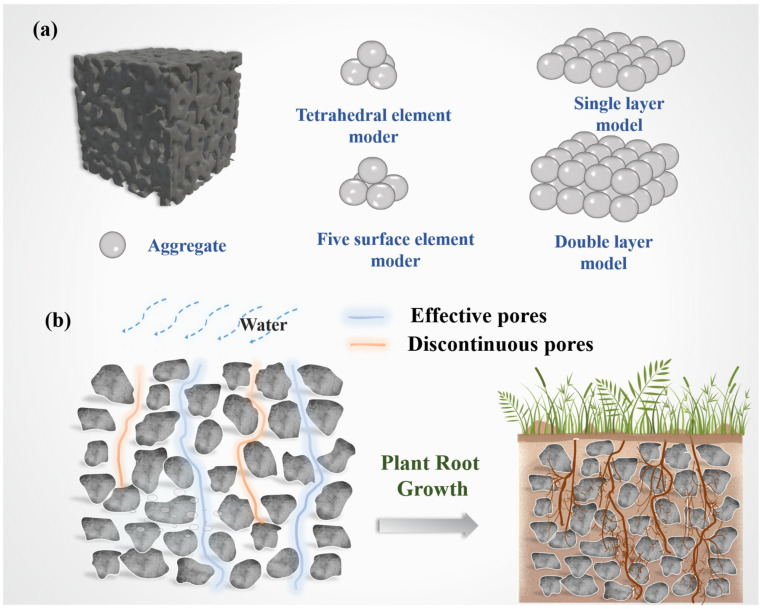
(**a**) Geometric packing model of aggregate particles; (**b**) functional partitioning of connected and discontinuous pores with schematic illustration of root accessibility.

EPC employs a coarse aggregate skeleton as its primary load-bearing component, where the aggregates form a self-stabilizing structure through multi-point contact, with the cement paste serving only to coat and bond the particles. Its pore structure is directly determined by the geometric arrangement of aggregate packing, representing a structural mechanism distinctly different from conventional concrete. The packing pattern of aggregate particles can be described using sphere packing theory (Figure 7a). The achievement of target porosity and compliance with strength design requirements largely depend on whether the coarse aggregates are effectively enveloped by cement paste to form stable, continuous voids [23].

**Figure 8:**
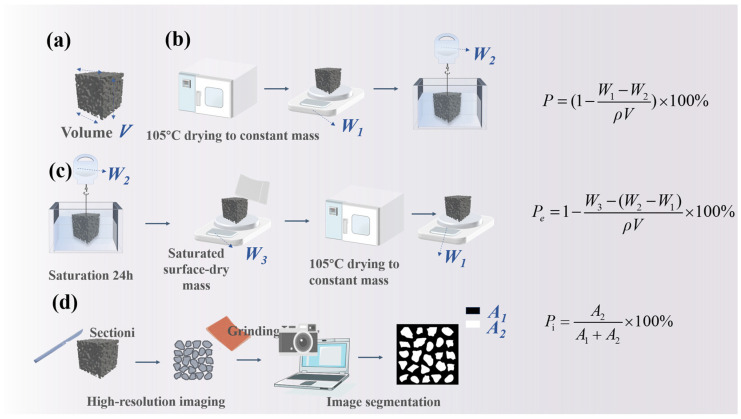
Schematic of simple test methods for *P* and *P_e_*: (**a**) specimen volume measurement; (**b**) *P*; (**c**) *P_e_*; (**d**) porosity determination by 2D image analysis.

To distinguish the actual efficiency of pores, two indicators are commonly used in research: *P* and *P_e_* (Figure 7b). *P* encompasses all pore spaces within the concrete, including both interconnected pores and isolated, discontinuous pores. In contrast, *P_e_* refers only to the proportion of pores connected to the external environment and capable of participating in fluid transport, more accurately reflecting actual service performance such as water permeability, air permeability, and root penetration [92]. Due to the presence of discontinuous pores, the *P_e_* of EPC is typically lower than the *P*. Figure 8 illustrates common testing methods for total *P* and *P_e_* (volume method, underwater weighing method) and the porosity calculation using 2D image analysis. In mechanical performance prediction, introducing effective porosity instead of total porosity can significantly improve the accuracy of compressive strength calculations [93].

**Figure 9:**
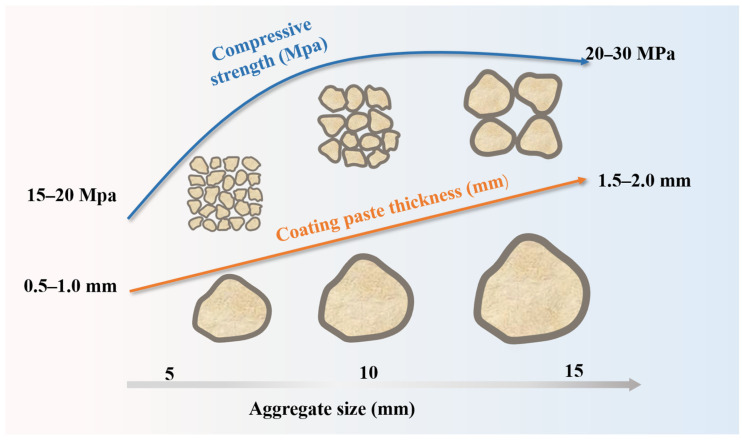
Schematic illustration of cross-scale correlations between aggregate particle size, CPT, and compressive strength of EPC.

Aggregate particle size governs the balance between pore connectivity and mechanical performance by modulating the coating paste thickness (CPT). As illustrated in Figure 9, increasing aggregate size linearly increases CPT and initially enhances compressive strength. However, beyond an optimal range, strength stabilizes as excessive paste thickness diminishes the constraining effect on the aggregate skeleton [94]. Sufficient paste coverage is required for robust ITZ bonding, yet excessive densification compromises the permeability essential for ecological function. Consequently, moderate aggregate sizes achieve the optimal CPT that reconciles microscale interfacial strength with mesoscale pore connectivity [95]. Consistent with this mechanism, Chen et al. [96] discovered through slice layering and digital image processing that with the same w/b ratio, larger aggregate particle sizes result in higher *P_e_*. Wang et al. [20] further revealed through CT reconstruction that pores formed by larger aggregates exhibit higher connectivity and lower *τ*, but the increased pore throat size leads to a looser structure. While the characteristics of highly connected large pores benefit water permeability and root extension, they reduce the constraining effect of paste encapsulation [30].

### 4.2. Pore Space Structural Characteristics: τ, Pore Throat, and Three-Dimensional Networks

**Figure 10:**
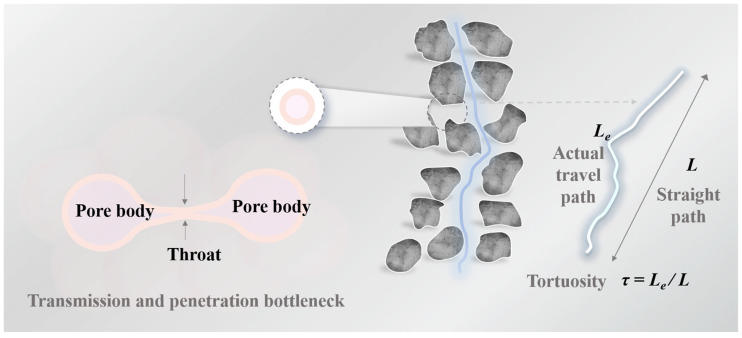
Conceptual model of pore throat structure and τ.

*P_e_* only characterizes the volume proportion of connected pores. However, EPC with the same *P_e_* may exhibit significantly different transport properties. Specifically, the permeability coefficient can vary by 3–5 times, and root penetration resistance may differ by an order of magnitude. This discrepancy arises because fluids and roots do not travel in straight paths through the pore network but instead navigate through tortuous channels constrained by narrow pore throat structures. Therefore, analyzing *τ*, pore throat size, and three-dimensional connectivity characteristics is key to understanding the performance mapping of mesostructures [97]. Figure 10 illustrates the conceptual model of pore throat structure and *τ*.

#### 4.2.1. *τ*

As shown in Figure 10, *τ* is defined as the ratio of the actual path length traversed by fluid or roots to the straight-line thickness of the specimen. In EPC, *τ* has dual physical significance: From a hydraulic perspective, *τ* directly determines the flow resistance; according to the Kozeny–Carman (K–C) model, the permeability coefficient is inversely proportional to *τ^2^* [98]. From a biological perspective, high *τ* implies that roots must follow tortuous paths, which increases mechanical friction and penetration energy expenditure while simultaneously prolonging water retention time within pores, thereby benefiting water conservation and root supply during drought periods [99]. The K–C model and its modifications for predicting EPC permeability are summarized in Table 2.

The predictive models surveyed above differ markedly in their underlying assumptions and applicability to EPC. For permeability, the classical K–C model assumes idealized parallel capillaries and employs *P* and specific surface area (*S*) as inputs; while conceptually tractable, it tends to underestimate permeability for EPC because isolated pores are neglected [98]. Modified versions incorporating *P_e_* and weighted tortuosity significantly improve prediction accuracy (R^2^ = 0.90–0.98) for high-porosity (>20%) heterogeneous structures, yet their empirical constants require calibration for each aggregate–paste system [100,101]. The relative aperture correlation model establishes a linear relationship between τ and the relative mean pore size, but its applicability is limited to single-sized aggregate systems where pore geometry is well characterized [100].

For *τ* determination (Table 3), the Electrochemical impedance spectroscopy (EIS) method enables rapid laboratory assessment (*τ* = 1.28–3.45) but relies on equivalent circuit models that often assume a uniform pore solution distribution, an assumption that may be violated in partially saturated or matrix-filled EPC, leading to potential inaccuracies [102,103]. CT image tracing provides direct 3D visualization (*τ* = 1.59–2.41) with superior reliability [104,105], though at higher cost and lower throughput. Simplified geometric or empirical models, while offering theoretical or computational convenience, often fail to capture the complex and irregular void morphologies that are characteristic of EPC [22].

#### 4.2.2. Pore Throat

Pore throats are relatively narrow channels connecting adjacent pore bodies, often simplified as cylindrical throats linking spherical pores in three-dimensional network models [105]. Figure 10 presents a schematic of simplified pore throat structures [104]. Statistical analysis shows that pore throat cross-sectional areas of EPC are primarily distributed between 0 and 40 mm^2^, with lengths ranging from 0 to 40 mm. This size distribution significantly influences critical behaviors of the permeability coefficient and root penetration [107]. Hydraulically, pore throats act as bottlenecks: their size determines the critical pressure required for fluid entry. Smaller pore throats enhance capillary blocking, which benefits water retention but hinders rapid drainage; conversely, larger pore throats increase permeability coefficients but reduce water retention capacity during drought periods [22]. Mechanically, pore throats serve as barriers to root extension. When pore throat diameters are smaller than root diameters, roots must rely on root pressure or chemical dissolution to break through, resulting in significantly increased penetration resistance. In contrast, sufficiently large pore throats (typically exceeding several millimeters) allow herbaceous plant roots to penetrate directly through mechanical means. Consequently, root accessibility is governed by pore throat size distribution rather than average pore size.

### 4.3. Fluid Transmission Efficiency

#### 4.3.1. Water Permeability

**Figure 11:**
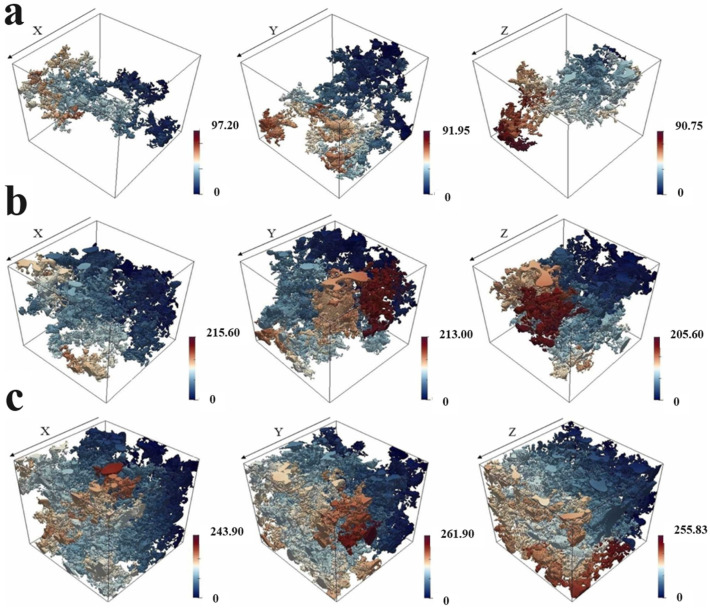
Initial infiltration paths in pore network models of EPC with different *P*: (**a**) 15% (*τ* = 1.22–1.62); (**b**) 20% (*τ* = 1.19–2.69); (**c**) 25% (*τ* = 1.45–2.91) [109].

The permeability coefficient is the most direct macroscopic manifestation of the mesoscopic pore structure. The traditional K–C model indicates that high *P_e_* can significantly enhance water permeability, yet the influence of *τ* on permeability behavior should not be overlooked [44]. Zhang et al. confirmed through CT reconstruction combined with CFD simulation that when *P* increases from 10% to 26%, the permeability coefficient increases by approximately 2.5 times and the streamline distribution is significantly strengthened [108]. Shan et al. further revealed that as *P* increases from 18% to 24%, *τ* decreases, the number of seepage channels increases, and connectivity improves, synergistically promoting permeability enhancement [107]. In addition, Zhang et al. characterized the evolution of pore network connectivity based on a Gaussian random field model [109]. As shown in Figure 11a, at *P* = 15%, the seepage paths are sparse and fragmented, with a low proportion of effectively connected pores. At *P* = 20%, continuous seepage channels increase significantly, and seepage efficiency improves (Figure 11b). At *P* = 25%, the pore network exhibits extensive interconnected characteristics throughout the domain (Figure 11c). Although *τ* in this model increases with *P*, the significant expansion of the connected domain still benefits the overall seepage capacity. Collectively, these studies suggest that the gains in permeability coefficient from increasing *P* generally outweigh any adverse effects of tortuosity variation. Additionally, pore throat size is also crucial. While small pore throats can enhance capillary water retention, they may hinder rapid drainage; when pore throats exceed 2 mm, they facilitate both water permeability and root penetration. In special aggregate systems, such as the EPC prepared with coral waste and seawater, the permeability coefficient can reach 1.29–2.65 cm/s due to the reinforced ITZ, significantly higher than the requirement for conventional EPC (≥0.1 cm/s), demonstrating excellent transport properties [43]. Appropriate permeability can mitigate the loss of matrix and nutrients under runoff conditions, providing a stable environment for plant growth [110].

#### 4.3.2. Mechanical Properties

Pores represent the weak links in concrete. Studies indicate that compressive strength is negatively correlated with *P_e_* [93]. It should be noted that while *P_e_* is more critical for transport properties, *P* exerts a more direct weakening effect on compressive strength, as isolated pores also serve as non-negligible stress concentrators. Pore geometry is equally important: flake-like pores are prone to stress concentration (with concentration factors reaching 3–5), whereas spherical pores cause less strength degradation. Therefore, optimizing aggregate gradation to obtain rounded, dispersed pores enables maintaining strength while ensuring adequate *P* [111].

Table 4 summarizes the commonly used models relating compressive strength to pore structure parameters. Regarding compressive strength, the exponential decay model is frequently employed to characterize the rapid strength reduction at elevated porosities [112]. The logarithmic model fits well over the typical EPC porosity range (15–30%) with R^2^ > 0.90 [113]. While a linear relationship may be assumed for convenience, it is often insufficient to capture the nonlinear decay in strength across the full porosity range typical of EPC [114]. Other models, such as those incorporating mean pore diameter and aggregate particle size, offer a more direct link to mix design parameters, particularly for single-sized aggregate systems [93]. More comprehensive statistical models, which integrate parameters including mean free spacing, pore diameter, specific surface area, and 3D pore distribution density, can achieve higher predictive accuracy while requiring more complex characterization [111].

#### 4.3.3. Prevention and Control of Plant Root Penetration and Blockage

The mesoscale pore throat size determines root accessibility. As previously demonstrated, the equivalent diameter of pore throats is significantly larger than the diameter of herbaceous plant fibrous roots (typically 0.2–1 mm). When the pore throat diameter exceeds that of the root, roots can mechanically penetrate directly. If pore throats are too small or have excessive *τ*, roots must rely on root pressure or chemical dissolution to break through, resulting in hindered growth. Therefore, the pore throat size distribution better reflects root accessibility than average pore size [50,57].

**Figure 12:**
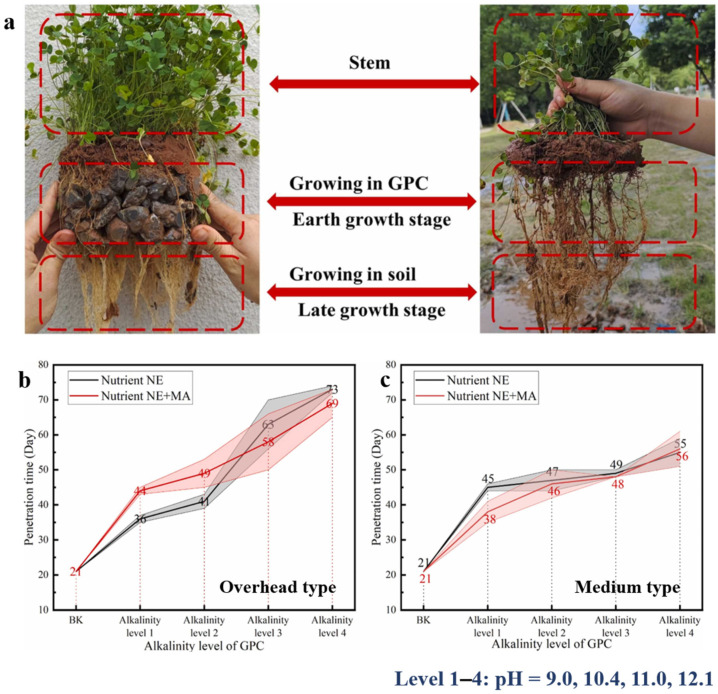
Schematic illustration of plant–concrete interactions: (**a**) root growth morphology of *Trifolium* in grass-planting concrete (GPC, i.e., EPC in this paper); (**b**) time required for root penetration through surface-covered GPC; (**c**) time required for root penetration through internally filled GPC [117].

Mesoscale pore throats provide physical channels, yet high alkalinity (pH > 11) inhibits root penetration. Before penetrating 7 cm concrete, *Trifolium* roots were restricted (31% of control length), but recovered by 3.4 and 4.6 times in length and forks after entering the soil (Figure 12a). Penetration time increased from approximately 40 days to over 65 days as pH rose from 9.0 to 12.1 (Figure 12b,c), with the overhead type delaying more than the medium type. This indicates cross-scale constraints of pore throat size and alkalinity; medium type reduces time by 15–20% through internal filling, mitigating clogging risks.

During service, sediment, small stones, and decomposed root systems are the primary sources of clogging. Zhou et al. [115] confirmed through CT scans that when the ratio of clogging particle size to pore throat diameter ranges from 0.6 to 0.8, the permeability coefficient decreases most significantly (clogging rate of 43–54%), forming stable bridging clogging. F-T durability is also related to pore throat size: smaller pore throats (capillary pores) are prone to stress concentration due to ice crystal expansion at low temperatures, while maintaining sufficiently large pore throats (typically > 2 mm) can provide space for ice crystal growth, mitigating frost damage [90,116].

## 5. Macro-Scale Pore Structure

The macro-scale focuses on the overall concrete components (such as ecological slope protection blocks and permeable pavement layers) and the pore systems formed by aggregate skeleton structures (typically referring to pores with diameters > 10 mm or interconnected pores formed by coarse aggregate accumulation). Their structural morphology directly determines the material’s overall engineering performance and ecosystem service functions, serving as a critical bridge connecting micro- and mesoscale structures with practical engineering applications.

### 5.1. Formation Mechanism and Controlling Factors of Macropores

The quantity, size, and connectivity of macropores are primarily determined by the particle size, gradation, and packing arrangement of coarse aggregates, serving as core control indicators for the macrostructural design of EPC. Theoretical frameworks such as the maximum density curve theory, particle interference theory, and fractal theory provide a theoretical basis for the design and prediction of target porosity [118]. The essence of these theories lies in quantitatively regulating target porosity by adjusting the volume ratio between aggregates and paste (*V_p_*/*V_s_*). By comparing the applicability of three gradation theories in describing aggregate packing states in EPC, it is found that the maximum density curve theory and particle interference theory are more suitable for preparing EPC with narrow particle size ranges. Based on these theories, a *P* prediction formula with aggregate particle size as the parameter can be derived, providing support for the quantitative design of macropore structures [103].

**Figure 13:**
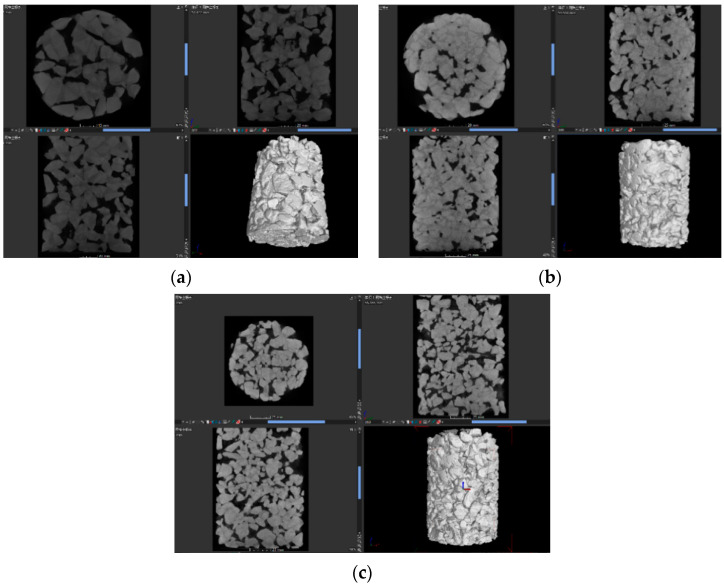
3D CT reconstruction of macropore structure in EPC: (**a**) *P* = 20%, aggregate 5–25 mm; (**b**) *P* = 30%, aggregate 5–25 mm; (**c**) *P* = 30%, aggregate 16–25 mm [20].

As shown in Figure 13, CT scans reveal that aggregates of different sizes create distinct pore zones within the component. Large aggregates form pores suitable for drainage and root penetration, whereas small aggregates form pores that aid water retention [20]. This heterogeneous distribution is the key to macro-scale design: by properly combining different aggregate sizes, rapid drainage and long-term water retention can be achieved simultaneously within a single component [47,48,49]. Therefore, the essence of macro-scale design lies in constructing appropriate macropore structures through aggregate system design based on specific engineering needs (such as slope protection, pavement, or wetland restoration).

### 5.2. Ecological Function Realization

**Figure 14:**
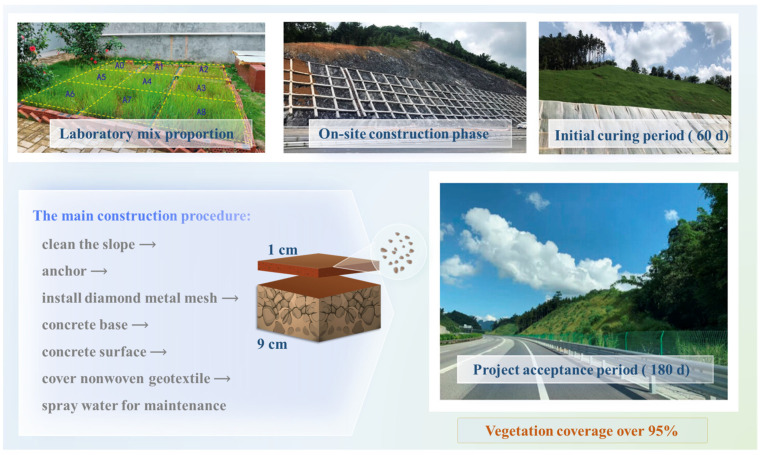
Construction process and vegetation restoration sequence of EPC [119].

Interconnected macropores serve as the fundamental medium for EPC to realize its ecological functions, playing an irreplaceable role in vegetation growth, hydrological cycling, water purification, and microclimate regulation. As shown in Figure 14, Chang et al. constructed interconnected skeletal pore networks via layered spraying. Herbaceous roots penetrated within 60 days, and a stable shrub–grass composite community with 95% coverage was established by 180 days. This outcome verifies the supporting role of macropore structures in plant growth [119]. Table 5 summarizes the recommended macropore design parameters for various EPC applications, tailored to specific ecological functions and engineering requirements.

#### 5.2.1. Plant Growth Space Support

The interconnected macropores, ranging from millimeters to centimeters, provide essential space for root penetration and elongation. Pore size, connectivity, and *P* directly determine root depth, distribution density, and plant survival rate. Excessively large pores may lead to substrate instability under rainfall erosion, while overly small pores hinder root penetration and growth. Engineering practices demonstrate that EPC with an interconnected porosity of 25–30% and an average pore size of 2–3 mm can effectively meet plant growth requirements [124].

#### 5.2.2. Hydrological Regulation and Runoff Control

**Figure 15:**
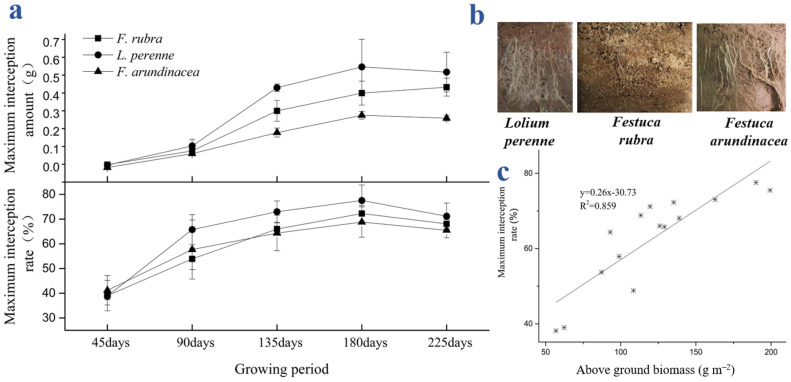
The canopy interception characteristics and root-canopy synergistic hydrological regulation mechanism of EPC. (**a**) Dynamic changes in maximum interception capacity and interception rate of three herbaceous plant canopies during the growth period; (**b**) root morphological characteristics of different species; (**c**) linear correlation between aboveground biomass and maximum interception rate [52].

Macropores provide primary channels for rapid rainwater infiltration, while the synergistic interaction between vegetation canopy and root systems significantly enhances surface runoff regulation [10]. As shown in Figure 15a, the canopy interception capacity of three herbaceous species increased substantially with growth period, with *L. perenne* achieving a maximum interception rate of 77.5% at 180 days, effectively reducing rainfall kinetic energy and delaying surface runoff generation. Concurrently, the well-developed root systems (Figure 15b) penetrate the EPC matrix, improving substrate permeability and structural stability while complementing the infiltration function of macropores. The strong linear correlation between aboveground biomass and interception rate (R^2^ = 0.859, Figure 15c) indicates that appropriate species selection combined with optimal growth periods (90–180 days) maximizes the interception–infiltration synergy of EPC. This approach effectively restores hydrological connectivity between terrestrial and aquatic environments in coastal wetlands and riverbank slopes, mitigating the water cycle disruption and habitat fragmentation caused by traditional hardened revetments [52]. However, most existing studies rely on short-term tests, with limited long-term field monitoring data on the hydrological performance of EPC. In addition, root vitality varies seasonally, and the preferential flow paths formed by root decay may alter water transport. Yang et al. demonstrated that decayed roots cause water-holding pore collapse, and rotten root pores form new preferential paths, leading to hydraulic deterioration [125]. Environmental factors such as F-T cycles can also affect long-term infiltration capacity, as studies have shown that unmodified EPC experiences significant permeability loss after 160 F-T cycles [126].

#### 5.2.3. Water Purification Effect

**Figure 16:**
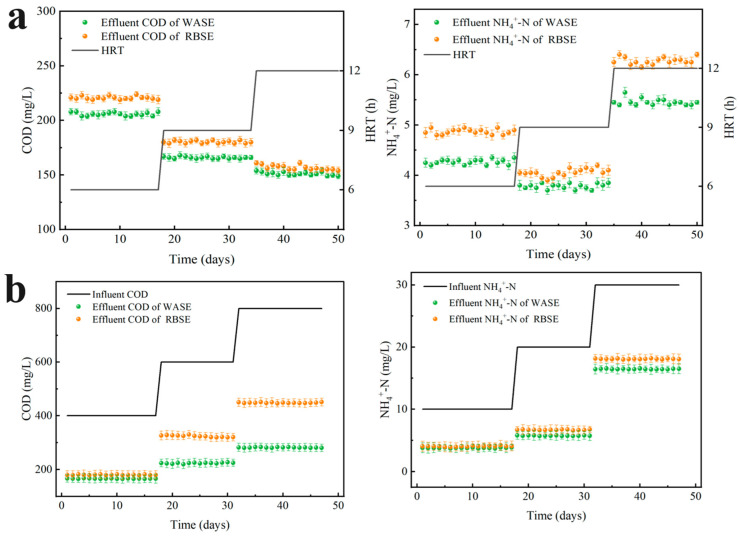
Removal of COD and NH_4_^+^–N by EPC-based purification system: (**a**) at different HRT; (**b**) under different influent concentrations [130].

Macropores and internal fillings of ecological EPC provide favorable habitats for microbial attachment. Macropores function not only as transport channels but also offer extensive internal surface areas crucial for biofilm formation. Pollutant removal is achieved through a triple mechanism involving physical filtration, chemical precipitation, and biodegradation [127]. Specifically, the porous structure filters and adsorbs suspended solids from runoff, while Ca^2+^ and Mg^2+^ ions released from the cementitious matrix react with NH_4_^+^ and PO_4_^3−^ to form precipitates. Concurrently, enriched microbial communities within the pores efficiently degrade organic pollutants, significantly enhancing water purification efficacy [128]. Xie et al. employed biochar-modified EPC and achieved TN and TP removal rates of 89.4% and 82.0%, respectively, significantly outperforming the unmodified control group, confirming that moderate pore filling can enhance biological purification efficiency without compromising permeability [129]. Zhang et al. constructed a purification system with EPC as the core, and found that when the hydraulic retention time (HRT) was 9 h, the removal rates of COD and NH_4_^+^–N reached 62.67% and 71.21%, respectively (Figure 16a). Meanwhile, the system maintained stable removal performance under different influent concentrations, indicating that EPC itself possesses good water purification capability and environmental adaptability (Figure 16b) [130]. Furthermore, EPC prepared with recycled aggregates (e.g., construction waste, pumice) demonstrates effective removal of TSS, TN, TP, NO_3_^−^–N, COD, and NH_4_^+^–N from stormwater runoff, exhibiting significant potential for non-point source pollution control [131].

The same microbial activity that enables water purification may also induce localized deterioration over extended service periods. Microorganisms colonizing pore surfaces can produce biogenic acids through their metabolic activities, progressively reducing the alkalinity of the cement matrix and dissolving hydration products [132]. Biofilm formation on pore walls may also facilitate the ingress of aggressive ions and accelerate degradation at the ITZ [133]. These negative effects are generally slower than chemical attack and are often outweighed by the ecological benefits in well-designed EPC systems, but they should be considered in long-term durability assessments, particularly for applications involving continuous water exposure or nutrient-rich runoff.

#### 5.2.4. Improvement of Urban Microclimate and Carbon Sequestration Through Greening

The synergistic interaction between macropore structure and vegetation cover reduces surface temperature through transpiration, and combined with the high permeability and reflectivity of the material itself, effectively alleviates the urban heat island effect [24]. Meanwhile, vegetation photosynthesis sequesters atmospheric CO_2_, enhancing terrestrial carbon sink capacity and providing an ecological pathway toward carbon neutrality goals. Furthermore, optimization of macropore structure can increase microbial abundance and diversity, strengthen ecosystem stability, and provide theoretical support and design insights for ecological restoration [134].

### 5.3. Engineering Safety Performance

EPC must possess sufficient structural stability, scouring resistance, and load-bearing capacity while fulfilling ecological functions, ensuring engineering safety and long-term service performance.

#### 5.3.1. Slope Stability and Root Reinforcement Effect

**Figure 17:**
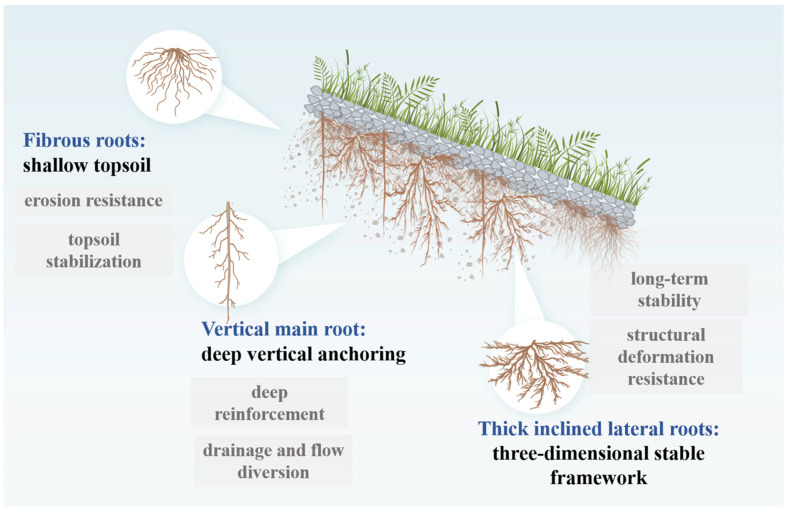
Functional schematic of different root architectures in EPC for slope protection.

EPC slope protection maintains stability through self-weight, base interface friction, root reinforcement, and anchoring effects. Macropores serving as rooting space directly determine root penetration depth and distribution patterns, thereby influencing overall reinforcement effectiveness [124]. A well-designed macropore structure enables roots to integrate with the concrete matrix, significantly enhancing slope resistance to sliding and deformation [135]. As shown in Figure 17, fibrous roots form dense shallow networks against erosion, taproots provide deep anchoring through vertical penetration, and thick roots establish stable frameworks for long-term stability. Species selection should thus be tailored to specific slope stability requirements [125].

#### 5.3.2. Scour Resistance Performance

Macro-scale surface texture and internal pore structure reduce runoff velocity, dissipate flow energy, and enhance substrate scour resistance [136]. Studies indicate that incorporating additives such as polyacrylamide (PAM) and palm fiber optimizes matrix structure, strengthens inter-particle cohesion, and significantly reduces runoff generation and soil loss. PAM promotes water-stable aggregate formation through cationic bridging, while palm fiber provides mechanical reinforcement. In contrast, biochar may reduce particle density and inter-particle contact area, weakening cohesion and potentially compromising scour resistance [137]. The efficacy of these amendments also depends on their molecular weight, dosage, and the textural properties of the substrate. However, amendments like biochar may compromise scour resistance by reducing particle adhesion, necessitating additive selection based on macro-scale structural characteristics. As the planting substrate, its scour resistance and nutrient retention capacity directly influence the efficacy of ecological restoration for exposed slopes [138,139]. The anti-scouring performance of EPC is further modulated by construction parameters and the progressive establishment of vegetation. For instance, construction parameters such as perforation design and placement thickness may influence the erosion resistance of EPC. The effectiveness of additives such as PAM and palm fiber also varies over time; although they can reduce initial runoff time by over 46% and achieve up to 94% sediment reduction, these benefits may diminish as vegetation establishes [137]. Meanwhile, the addition of super absorbent polymer can enhance the scouring resistance of planting soil by 68.9%, yet the long-term durability of such amendments under repeated rainfall events remains insufficiently studied [30]. These observations suggest that the anti-scouring performance of EPC may be more appropriately considered as a time-dependent system property rather than a static material attribute.

#### 5.3.3. Mechanical Performance and Engineering Applicability

As paving or revetment materials, the macropore structure determines load transfer paths and stress distribution through the synergistic interaction of aggregate interlocking and paste encapsulation [140]. The self-stabilizing skeleton formed by point contact of coarse aggregates, combined with hardened cement paste, constitutes a three-dimensional load-bearing network that satisfies pedestrian and light traffic loading requirements (compressive strength: 10–15 MPa) while providing energy dissipation and vibration damping characteristics [141]. Typical EPC design parameters comprise a porosity of 20–35% and an average pore diameter of 5–10 mm, balancing structural safety with the spatial requirements for ecological functions [10]. As plant roots penetrate and the substrate fills, a root-soil–concrete composite gradually develops, enhancing overall load-bearing capacity and deformation resistance through root reinforcement in later stages [142]. This makes EPC suitable for ecological parking lots, landscape walkways, and low-velocity riverbank protection applications.

#### 5.3.4. Plant Selection and Engineering Application

**Figure 18:**
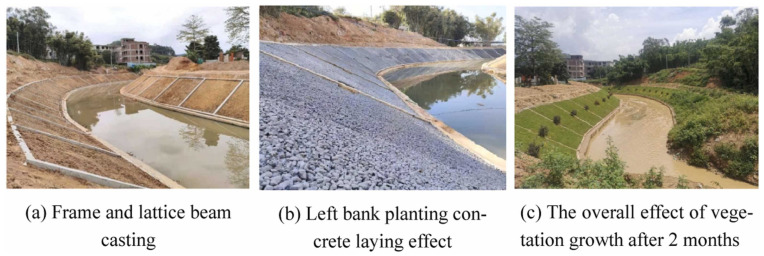
Construction progress of river slope protection [23].

Plant selection is critical to ensuring the long-term serviceability of vegetation concrete. Priority should be given to native species exhibiting strong vitality, well-developed root systems, and tolerance to saline-alkali conditions, drought, and high pH, as well as broad environmental adaptability. *Festuca arundinacea*, *Lolium perenne*, and *Cynodon dactylon* have been extensively employed in ecological revetment projects worldwide, offering advantages including high survival rates, extended greening periods, and low maintenance costs (Table 6). Engineering applications demonstrate that appropriately selected species can establish stable growth without long-term artificial maintenance, synergistically achieving the dual objectives of ecological restoration and bank protection in conjunction with the concrete matrix (Figure 18) [143,144].

## 6. Comprehensive Analysis of Multi-Scale Pore Structures

**Figure 19:**
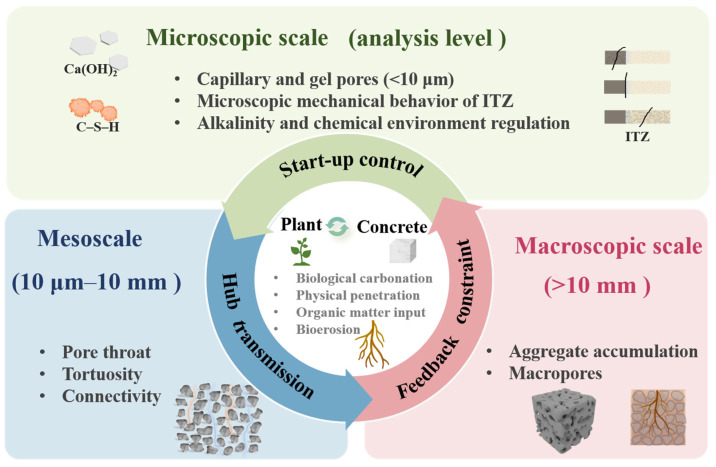
Cross-scale synergy of multi-scale pore structures in EPC.

The multi-scale pore structure of EPC does not exist in isolation. Close, synergistic, and constraining relationships exist among the micro-, meso-, and macro-scale structures, which jointly determine the integrated performance of the material. The microscale establishes the foundation for matrix strength and alkaline environment, the mesoscale governs mass transport efficiency and damage evolution pathways, while the macro-scale directly delivers ecological functions and engineering safety [150,151,152]. These three scales interact through reciprocal feedback, forming a closed-loop coupling system characterized by microscale initiation and control, mesoscale hub transmission, and macro-scale feedback constraints. As shown in Figure 19, the chemical barrier and strength foundation at the microscale, and pore throats, *τ*, and connectivity at the mesoscale determine the physical resistance to root penetration and water transport efficiency; aggregate accumulation and macropores at the macro-scale provide growth space and mechanical skeleton for plant roots. The interaction among plants, soil, and concrete serves as the medium for cross-scale coupling, organically integrating the microscale chemical environment, mesoscale transport characteristics, and macro-scale ecological functions. A thorough understanding of this cross-scale mechanism is the key scientific prerequisite for reconciling the inherent conflict between high ecological demands and high structural requirements in ecological concrete, thereby achieving synergistic performance optimization [153,154].

### 6.1. Cross-Scale Interactions

The microscale serves as the fundamental basis for EPC performance. ITZ compactness determines interfacial bonding stability and constitutes the microstructural basis for macroscopic strength [37,44]. The distribution of C–S–H gel pores and capillary pores directly influences the constitutive behavior of the matrix and the damage initiation threshold [47,48,49]. Meanwhile, pore solution alkalinity fundamentally determines root viability [76,77]. Studies indicate that microcracks generated in the ITZ during early hardening due to shrinkage stress, even at microscopic scales, can act as nucleation sites for subsequent macroscopic failure [42].

The mesoscale serves as the critical intermediary bridging micro- and macro-scale behaviors. The interconnected pore network formed by aggregate accumulation provides transport pathways for water, nutrients, and deleterious ions, while simultaneously acting as the conduit for microscale damage propagation toward macroscopic deterioration. Key parameters, including effective porosity, *τ*, and pore throat dimensions, directly govern fluid transport efficiency and root penetration space [99]. Furthermore, mesoscale structure dictates whether microscale damage is amplified or suppressed: when microcracks propagate into pore throats, favorable geometries may induce crack blunting or deflection, whereas sharp throats with stress concentration facilitate rapid crack coalescence, leading to macroscopic failure [93,155].

The macro-scale constitutes the ultimate level of performance realization. Macropores formed by the aggregate skeleton provide growth space for plant roots and primary pathways for rainwater infiltration, while also fulfilling engineering functions of load transfer and slope stabilization [156,157]. Macroscopic structure not only directly delivers ecological and engineering services but also transmits external environmental loads, such as scouring, F-T cycles, and ion attack, back to the meso- and microscales, thereby driving structural evolution [124,158].

### 6.2. Performance Trade-Off and Synergistic Optimization Mechanism

The core scientific issue of EPC lies in the conflict between high ecological requirements and structural demands, which essentially manifests as cross-scale pore constraints. To ensure water permeability and vegetation growth, macroscopic large pores and mesoscopic high connectivity are required, yet this weakens the mechanical framework and intensifies microscopic stress concentration. Conversely, to enhance strength and durability, microscopic densification and minimal mesoscopic defects are needed, but this may inhibit plant growth.

**Figure 20:**
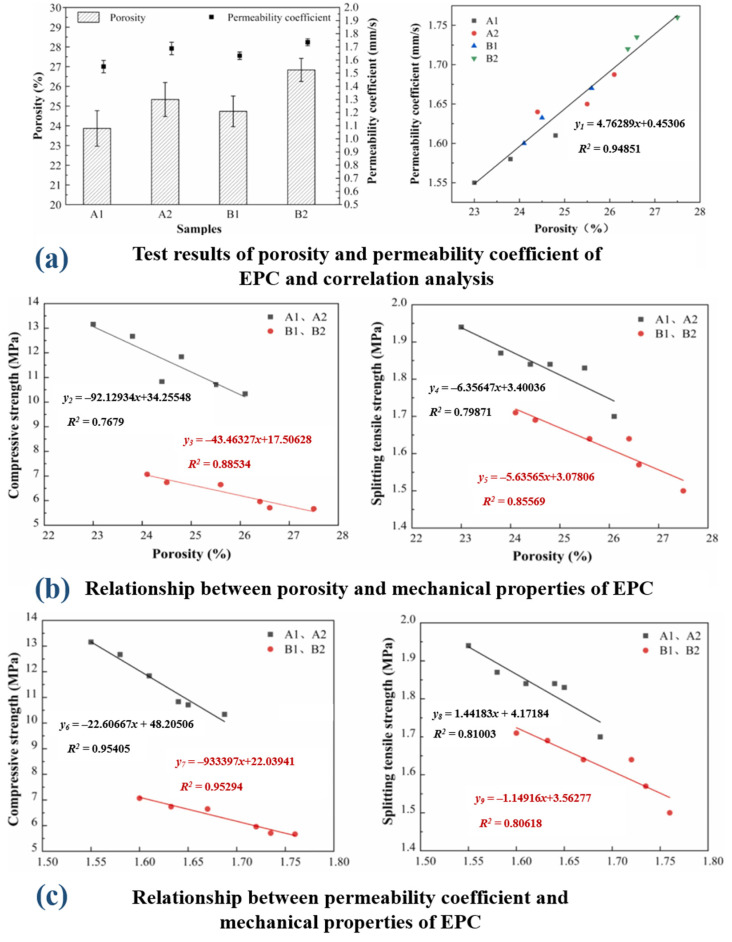
Trade-off of *P*, permeability coefficient and mechanical properties of EPC. (**a**) *P* and permeability test results and their correlation; (**b**) Correlations between porosity and mechanical properties; (**c**) Correlations between permeability coefficient and mechanical properties [65].

As shown in Figure 20, although merely increasing mesoscale connectivity can linearly enhance permeability, it significantly compromises compressive strength. However, through microscale modification via the diatomaceous earth–oxalic acid–ferrous sulfate composite system, optimizing hydration products and reducing alkalinity, an 85–97% improvement in compressive strength can be achieved while maintaining 20–30% macro-scale porosity and appropriate meso-scale pore throat structures [65]. This demonstrates that performance breakthroughs require synergistic optimization across the micro-, meso-, and macro-scales rather than single-scale adjustment. Table 7 summarizes the typical values, key sources of variability, and engineering implications for the main physical and mechanical properties of EPC reported in the literature. Notably, deviations from the optimal ranges, whether excessive or insufficient, can compromise either structural stability or ecological functionality.

Existing literature has proposed various optimization strategies to address this trade-off. Phuyal et al. [163] demonstrated that synchronous optimization of pore structures at the micro- and macro-scales through the incorporation of precipitated calcium carbonate and upcycled recycled concrete aggregate can significantly enhance the synergistic mechanical and ecological performance. Regarding cross-scale research on alkalinity control and strength balance, certain controversies exist: while most studies suggest that alkali reduction treatment causes 10–30% strength loss [158], Yang et al. [72] indicated that under a strictly controlled liquid-to-solid ratio, alkali reduction treatment not only fails to significantly impair mechanical properties but may even slightly improve durability. Furthermore, paraffin wax, as a physical alkali sealing material, causes negligible strength loss, while silane and epoxy resins can form surface barriers to rapidly isolate alkali migration without compromising substrate strength [76,77], providing diverse options for cross-scale chemical environment regulation. These conflicting findings reflect the sensitivity of alkali reduction outcomes to experimental conditions. Differences in the concentration and type of acidic agents, the duration of treatment, the liquid-to-solid ratio, and the initial pH and composition of the cementitious matrix all contribute to the observed variability. Furthermore, the evaluation methods for strength and durability differ across studies, with some using standardized curing conditions and others simulating field exposure scenarios, complicating direct comparisons.

Regarding the eco-engineering feedback mechanism, Zeng et al. [156] revealed the cross-scale synergistic protection mechanism of EPC: macro-scale leaf transpiration regulates moisture, mesoscale root anchoring reinforces soil, and microscale matrix pores retain water and supply nutrients. Shu et al. [157] further found that microorganisms (e.g., *Glomus mosseae*) can improve plant growth through microscale rhizosphere effects, complementing the regulatory role of biological factors in cross-scale material cycling.

**Figure 21:**
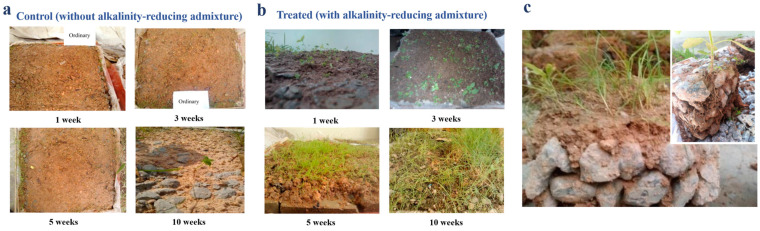
Comparison of vegetation growth and root penetration morphology before and after alkali reduction treatment. (**a**) High-alkalinity control group without alkali-reducing admixture; (**b**) vegetation growth status of low-alkali experimental group with alkali-reducing admixture at 1, 3, 5, and 10 weeks; (**c**) morphology of plant root penetration [164].

The ecological benefits of cross-scale synergistic optimization can be directly validated through vegetation performance. Bao et al. [164] conducted *Bahia grass* planting experiments on EPC incorporating a self-designed alkalinity-reducing admixture. As shown in Figure 21, the high-alkalinity control group without the admixture exhibited delayed seed germination and sparse growth, whereas the low-alkalinity treatment group grew rapidly within the first three weeks after sowing, formed a dense turf after ten weeks, and the roots could extend downward through the interconnected internal pores of the concrete. Chen et al. employed the superabsorbent polymer–oxalic acid–ferric sulfate composite alkali-reduction system to treat the EPC platform (with the pore solution pH reduced to 9.18 after 56 d). While maintaining a macro-scale porosity of 20–30%, the 30 d vegetation coverage was also significantly higher than that of the high-alkalinity control group [165]. This further indicates that the cross-scale synergy between microscale alkalinity regulation and macro-scale porous structure can create a suitable micro-environment for plant growth while ensuring structural bearing capacity, thus achieving simultaneous optimization of engineering protection and ecological greening.

The cross-scale framework reveals that EPC performance emerges from micro-, meso-, and macro-scale pore interplay, with the dominant constraint shifting across application scenarios. For vegetation-dominated applications, design must proceed from plant survival requirements, following the sequence of chemical compatibility, physical space, and geometric passage. High-alkaline environments inhibit root elongation and lateral formation, and interfere with uptake of calcium, iron, manganese, and boron; without adequate alkalinity control, roots cannot survive even when pores are physically accessible [117]. Macro-scale porosity then sets the upper limit for root penetration depth and water storage; root penetration is markedly hindered when specimen thickness exceeds 10 cm, with 6–10 cm recommended [166]. Mesoscale pore throat geometry finally determines whether roots pass through mechanically or must exert pressure and chemical dissolution, directly affecting establishment time [125]. This vegetation-centered sequence replaces the conventional porosity-centric approach with targeted, scale-specific intervention.

### 6.3. Cross-Scale Degradation Evolution Under Environmental Effects

The durability degradation of EPC under environmental actions typically follows a cross-scale pathway: external agents penetrate at the macro-scale, transport and diffuse at the mesoscale, and accumulate as microscopic damage reactions.

Taking ion attack as an example, chloride and sulfate ions first enter the interior through macro-scale macropores, a process controlled by surface porosity and opening aperture [78]; subsequently, ions migrate through the mesoscale network, with transport rates governed by *τ*, pore throat dimensions, and connectivity [99]; ultimately, ions reach the microscopic ITZ and react with hydration products (forming Friedel’s salt and ettringite), where volume expansion induces internal stress and initiates microcracking [79]. Initial microscopic damage does not directly cause failure [167]; however, under sustained loading coupled with environmental factors, damage propagates and interconnects through the meso-scale network, exhibiting cumulative amplification. Once damage density exceeds the critical threshold, rapid coalescence occurs, leading to accelerated degradation of macro-scale performance [81,168].

For ion attack, reducing mesoscale pore connectivity and increasing tortuosity through structural design can effectively suppress overall degradation to a certain extent, even when macro-scale porosity is relatively high [169]. For F-T cycles, optimizing microscale pore structure to provide stress accommodation space can effectively disperse ice expansion stress [116,170]. Therefore, accurately identifying and actively regulating (e.g., via numerical simulation) the bottleneck scale under different environmental actions is key to maximizing the durability of EPC.

## 7. Research Gaps and Future Directions

### 7.1. Limitations of Existing Research

(1) Rheological control of the casting process. The rheological behavior of fresh EPC mixtures significantly influences pore structure formation and uniformity. Excessive paste fluidity leads to paste pooling at the specimen bottom during casting, blocking interconnected pores. Insufficient fluidity prevents uniform aggregate encapsulation, causing particle detachment and severe strength reduction [171]. However, the quantitative relationship between paste rheology and the resulting pore structure remains poorly characterized. This uncertainty, combined with the lack of standardized test methods specifically designed for EPC, forces researchers to rely on extensive trial-and-error approaches to determine appropriate mixture workability for specific raw materials, hindering large-scale engineering applications [172].
(2) Multi-scale performance prediction and optimization. The mechanical, permeability, vegetative, and durability properties of EPC are governed by cross-scale pore structures. Traditional empirical models (e.g., linear regression, power-law fitting) are insufficient for capturing the complex nonlinear interactions among parameters across different scales [173,174]. While machine learning approaches have shown promise [21], their application remains limited by the scarcity of comprehensive datasets that systematically cover microstructural characteristics, mesoscopic pore network parameters, and macro-scale performance indicators. Consequently, mix proportion design remains largely empirical, with limited predictive capability for performance under varying service conditions.
(3) Pore micro-ecosystem response mechanisms. Existing studies have primarily examined the macroscopic dose-effects of cement matrix properties on seed germination and plant growth. The diversity, activity, and functional roles of soil microbial communities within concrete pores, as well as their interactions with plant roots, remain largely unexplored [175]. In particular, the combined effects of high alkalinity, limited organic carbon availability, and restricted pore space on microbial colonization and metabolic activity are still poorly understood, limiting the potential for biologically enhanced EPC performance [176].
(4) Capabilities for simulating and predicting cross-scale damage evolution remain inadequate. The degradation of EPC under environmental actions follows a cross-scale pathway from external action, through mesoscale transport and microscale damage, to macroscopic failure. Currently, computational models capable of bridging nanometers to centimeters are lacking, making precise prediction of long-term service performance difficult.

### 7.2. Future Research Directions

#### 7.2.1. Deep Learning-Driven Cross-Scale Intelligent Design

Complex nonlinear mapping relationships exist between the multi-scale pore structure and comprehensive performance of EPC, making accurate prediction and synergistic optimization difficult for traditional empirical models. Existing studies have preliminarily demonstrated the potential of deep learning and machine learning approaches. Li et al. [49] established an Ant Colony Optimization-Back Propagation Neural Network using 13 input parameters to predict compressive strength and porosity, achieving a coefficient of determination of 0.97 on the test set. Yu et al. [177] constructed machine learning models for the simultaneous prediction of permeability coefficient and compressive strength. Sathiparan et al. [178] employed XGBoost to predict the compressive strength of fly ash blended EPC using 437 datasets, achieving a test R^2^ of 0.95, with aggregate size identified as the most influential parameter. Regarding pore structure identification, Yu et al. [179] proposed an improved UNet for CT image pore segmentation, reducing the mean bi-directional Hausdorff distance by 48.7% to 72.4%. Wang et al. [180] employed Mask R-CNN to achieve synchronous quantitative segmentation and three-dimensional reconstruction of cement bridges and pores. Recent advances have extended deep learning-based segmentation to the full three-phase structure of EPC, including pore, aggregate, and paste phases, with Mask R-CNN achieving accurate identification of all three phases [181]. Transformer-based architectures have also been explored for CT image segmentation of multiphase composite building materials [182]. Dong et al. [21] developed an analytic hierarchy process–grey wolf optimizer–back propagation neural network model to simultaneously optimize four performance categories: mechanical properties, permeability, pollutant removal, and vegetation suitability. Future research should focus on constructing high-quality databases encompassing micro-, meso-, and macro-scale parameters, and developing physics-informed neural networks and interpretable analysis methods, to promote the transformation of EPC from empirical mix proportioning to cross-scale intelligent design [183].

#### 7.2.2. Integration of Microecosystem and Microbial Activity

Existing studies predominantly focus on the macroscopic effects of the cement matrix on plants, with limited investigation into the response and activity of soil microbial communities within concrete pores [184]. Microbial diversity and richness play crucial roles in maintaining ecosystem multifunctionality, including nutrient cycling, organic matter decomposition, and disease suppression. However, the high-alkaline environment and limited organic carbon within EPC may significantly inhibit microbial colonization and metabolic activity [185]. Future design should transition from a sole plant carrier to a synergistic habitat for plant-microbe communities, with specific research directions including screening alkali-tolerant growth-promoting strains and exploring their inoculation and survival strategies within concrete pores. Investigating how pore structural characteristics influence microbial migration, attachment, and biofilm formation. Evaluating the long-term synergistic effects of microbial-driven nutrient transformations such as nitrogen fixation and phosphorus solubilization on plant growth.

#### 7.2.3. Multi-Scale Computation and Intelligent Technology

To address the low efficiency and high cost of traditional empirical mix proportioning, future research should develop multi-scale numerical simulation systems based on real structural characterization. Drawing upon the multi-scale simulation approaches of Gong et al. [186], research should proceed from hydration kinetics through microscale pore structure evolution, ultimately coupling microscopic reactions with macroscopic mechanical behavior via pore mechanics to establish a cross-scale predictive chain. Meanwhile, combining X–CT with three-dimensional reconstruction techniques can accurately capture the real geometries of aggregate packing, pore connectivity, and root penetration, thereby constructing high-fidelity digital models spanning from mesoscale pore throats to macro-scale structural members and providing authentic structural boundaries for theoretical simulations [120].

Regarding intelligent construction and engineering applications, full life-cycle intelligent management technologies should be developed. Three-dimensional concrete printing (3DCP) offers an emerging pathway for fabricating EPC components with precisely controlled pore architectures, enabling the direct construction of designed pore networks without traditional formwork [187]. Digital twin technology offers a promising platform for integrating real-time monitoring data with predictive models to support lifecycle performance assessment [188]. During construction, real-time monitoring systems based on computer vision should be introduced to identify paste encapsulation uniformity and pore connectivity quality [189,190]. During service, wireless sensor networks (monitoring humidity, pH, strain, and root growth) should be deployed, combined with IoT to enable remote synergistic assessment of structural health and ecological performance. At the long-term scale, an integrated digital twin of material–plant–environment interactions should be constructed to simulate performance evolution under F-T, erosion, and vegetation succession, enabling predictive maintenance and whole-life-cycle management.

#### 7.2.4. Practical Implementation Roadmap

The cross-scale framework can be translated into a hierarchical implementation strategy that proceeds in the order of micro-, meso-, and macro-scales. At the microscale, chemical compatibility and interfacial bonding are first established. Alkalinity control (Section 3.3) serves as a prerequisite. OPC-based EPC should have its pore pH reduced to 7–9 prior to planting through SCM blending or secondary acid treatment, a range consistent with the tolerance of common slope protection species such as *Festuca arundinacea* and *Lolium perenne* (Table 6). Simultaneously, matrix densification and ITZ enhancement (Table 1) ensure effective root anchorage after penetration. At the mesoscale, aggregate gradation (Section 4.1) and paste volume control determine pore throat dimensions and connectivity. For herbaceous plants with root diameters of 0.2–1 mm, mechanical penetration is feasible when pore throats exceed 2 mm (Section 4.2.2). Below this threshold, roots rely on pressure or chemical dissolution, significantly prolonging establishment time. At the macroscopic scale, target porosity (*P* = 15–30%, Table 5) and specimen thickness (6–10 cm) are determined according to the root depth requirements of the target plants. Penetration is significantly hindered when thickness exceeds 10 cm (Section 6.2).

## 8. Conclusions

This review has examined the intrinsic correlations between the multiscale pore structure and comprehensive properties of EPC. The main findings are as follows.

(1) The mechanical, permeable, vegetative, and durable properties of EPC are influenced by the multiscale pore structure spanning from micro- to meso- and macro-scales. Traditional single-parameter indicators such as macro-scale porosity fail to fully characterize these complex performance attributes.
(2) The cross-scale mechanisms can be summarized as follows: the microscale characteristics of the ITZ and pore solution alkalinity serve as important controlling factors for strength development and vegetation compatibility; the mesoscale connected pore network plays a dominant role in fluid transport efficiency and erosion-induced degradation pathways; and the macro-scale skeletal pore structure contributes significantly to engineering performance and ecosystem service functions. The relative importance of each scale is context-dependent, varying with EPC type, intended application, environmental conditions, and testing methods.
(3) To achieve synergistic optimization of high ecological and structural requirements, it is necessary to consider the regulatory roles of micro- and mesoscale features in addition to macro-scale porosity. Specific measures may include comprehensive design approaches involving aggregate gradation, paste composition, pore morphology, and in situ alkalinity.
(4) Future research should focus on deepening the mechanistic understanding of plant-concrete and microbe–concrete interfaces, introducing multiscale numerical simulation, deep learning-based intelligent prediction, and smart construction monitoring technologies, thereby promoting the standardized, low-carbon, and large-scale application of ecological concrete in Sponge City construction, ecological revetment engineering, and slope restoration.

## Figures and Tables

**Table 1:** Microstructural optimization approaches and their mechanisms.

Optimization Strategy	Specific Measures	Mechanism	Main Effects	Ref.
Matrix densification	Ultrafine fillers (silica fume, nanoparticles)	Filling micro-/nano-pores	Refines harmful capillary pores, improves matrix density	[20,85]
Pozzolanic effect	Pozzolanic SCMs (fly ash, slag)	Secondary hydration with Ca(OH)_2_	Reduces alkalinity, strengthens interfacial bonding, refines pores	[86]
Paste stability control	Superplasticizers, rheology modifier	Optimized w/b ratio, minimized bleeding	Reduces microstructural defects caused by bleeding	[87]
Interfacial toughening	Polymer latex, synthetic fibers	Flexible ITZ transition layer, crack bridging	Inhibits microcrack propagation, enhances material toughness	[88,89]
Pore structure regulation	Aggregate gradation and paste-to-aggregate ratio	Optimizes pore size distribution while preserving macroporosity	Balances permeability-durability via optimal macro-/micro-pore distribution	[36,90]
Functional modification	Agricultural wastes such as straw biochar, wood chips	Alkali adsorption, energy dissipation	Assists dealkalization; rigid-flexible synergy under load	[75,91]

**Table 2:** The K–C model and its modifications.

Model Name	Mathematical Expression	Key Parameters	Applicable Features and Limitations	Ref.
Traditional K–C model	$K=\frac{P^{3}}{c\tau^{2}\cdot S^{2}\cdot (1-P)}\cdot \frac{\rho g}{\mu }$	*P*, *τ*, *S*	Based on the ideal parallel capillary assumption	[98]
Modified K–C model	$K=\frac{P_{e}^{3}}{c_{0}\tau^{2}\cdot S_{e}^{2}\cdot (1-P_{e})}\cdot \frac{\rho g}{\mu }$	*P_e_*, *S_e_*: effective specific surface area	Significantly improve prediction accuracy, with R^2^ reaching 0.90–0.98	[100]
Weighted *τ* correction model	$K=\frac{P_{e}}{c_{0}{\left(L_{e}/L\right)}_{w}^{2}\cdot {\left(SSA\right)}_{pe}}\cdot \frac{\gamma }{\mu }$	(*L_e_*/*L*)*_w_*: weighted *τ*	Applicable to high porosity (>20%) and heterogeneous pore structures	[101]
Relative aperture correlation	$\tau =a\cdot \frac{\bar{d_{p}}}{d_{a}}+b$ $K=\frac{P_{e}^{3}}{\tau^{2}\cdot {\left(1-P_{e}\right)}^{2}}\cdot \frac{\rho gd_{a}^{2}}{72\mu }$	$\bar{d_{p}}$: average pore size (calculated by LPF) *d_a_*: aggregate particle size	Establish a linear relationship between *τ*, and relative mean pore size (R^2^ = 0.995)	[100]

**Table 3:** Measurement methods and a summary of typical values of *τ*.

Determination Method	Fundamental Principles	Aggregate Particle Size (mm)	*P* (%)	*τ* Range	Applicable Scenarios	Ref.
EIS electrochemical method.	Based on the ratio of effective conductivity to pore fluid conductivity	2.36–9.5	15–30	1.28–3.45	Laboratory rapid testing	[99,102,103]
CT image tracking method	Perform 3D reconstruction of pore channels and calculate the ratio of actual path length to straight-line distance	4.75–9.5	17–27	1.59–2.41	Microstructure visualization	[104,105]
K–C equation inversion	Back-calculation from permeability test data.	1.19–4.75	20–26	1.07–5.03	When penetration data is available.	[106]
Simplified geometric model	Theoretical calculation of ideal sphere packing.			~1.414	Theoretical estimation	[22]

**Table 4:** Predictive models for the compressive strength–porosity relationship in EPC.

Model Type	Mathematical Expression	Key Parameters	Applicability and Accuracy	Ref.
Exponential decay model	$\sigma_{c}=\sigma_{0}e^{-bP}$	*P*, *σ*_0:_ theoretical strength at zero porosity	Classical form	[112]
Logarithmic model	$\sigma_{c}=72.9-18.4\ln(P_{e})$	*P_e_*	Applicable for porosity range 15–30%, R^2^ > 0.90	[113]
Linear model	$\sigma_{c}=-1.2863P+46.692$	*P*	Applicable only for narrow porosity range (14–23%)	[114]
Multi-parameter comprehensive model	$\begin{array}{l} \sigma_{c}=\alpha_{0}+\alpha_{1}\frac{\ln(d_{MFS})}{\ln(d_{n})}\\ +\alpha_{2}\frac{1}{S_{p}}+\alpha_{3}\ln(\Gamma_{3D}) \end{array}$	*d_MFS_*: mean free spacing, *d_n_*: number-averaged pore diameter, *S_p_*: specific surface area of pores, *Γ_3D_*: 3D pore distribution density	Highest accuracy; requires complex characterization	[111]
Aggregate-pore size correlation model	$\sigma_{c}=\sigma_{0}(1-mP_{e}){\left(\bar{d_{p}}/\bar{d_{a}}\right)}^{n}$	$\bar{d_{p}}$: mean pore diameter, *d_a_*: aggregate particle size	Applicable for single-sized aggregate systems	[93]

**Table 5:** EPC application scenarios and corresponding macropore parameters.

Application Scenario	Target Functions	Recommended *P*/Pore Size	Compressive Strength Requirement	Aggregate Size	Ref.
Ecological slope protection	Root anchoring, slope erosion resistance, ecological restoration	>25%/ 2–10 mm	5–10 MPa	10–25 mm	[6,8]
Permeable pavement (light load)	Rainwater infiltration, urban heat island mitigation, light-load bearing	15–25%/1.5–3 mm	15–25 MPa	5–10 mm	[96,120]
Sponge City sidewalk	Rainwater percolation, runoff reduction, pedestrian load	20–30%/ 2–8 mm	10–20 MPa	5–20 mm	[20,121]
Coastal wetland revetment	Tidal habitat provision, wave energy dissipation, salt resistance	20–35%/ 5–10 mm	10–15 MPa	10–30 mm	[28,122]
Water purification substrate	Nutrient (N/P) removal, microbial attachment, filtration	20–30%/ 3–8 mm	5–15 MPa	10–20 mm	[32,123]

**Table 6:** Plant species selection guide for EPC applications.

Plant Name	Plant Type	Root Characteristics	Pore Size	Stress Tolerance	Applicable EPC Environment	Ref.
*Festuca arundinacea*	Cool-season herb	Deep (tap + fibrous), penetrating 6–8 cm concrete	2–5 mm	Drought/cold tolerant; alkali resistant (pH 7–9)	General use; rapid establishment (3 d germination, 60 d coverage)	[9,52]
*Lolium perenne*		Fibrous (dense, shallow, high biomass)	2–5 mm	Not drought/saline tolerant	Temperate short-term cover; mixed sowing for erosion control	[25,145]
*Medicago sativa*		Taproot (deep, strong penetration)	5–10 mm	Highly drought/barren/alkali tolerant; N-fixing	Barren slopes; long-term restoration; N-source when mixed	[2,146,147]
*Cynodon dactylon*	Warm-season herb	Deep (stolons + taproot), trample-resistant	2–5 mm	Highly saline-alkali tolerant (pH > 11); not cold/drought tolerant	High-alkali (pH > 10) preferred; humid subtropical wetlands	[25,148]
*Zoysia japonica*		Fibrous (dense network, soil-binding)	2–5 mm	Highly drought tolerant; barren tolerant	Arid regions; low-maintenance Sponge City areas	[9,149]

**Table 7:** Variability of key physical and mechanical properties of EPC reported in the literature.

Parameter	Typical Value	Key Source of Variability	Implications of Deviation
*P*	15–30%	Aggregate gradation, compaction effort, w/c ratio	>30%: significant strength loss; <15%: inadequate permeability and root penetration [22,159]
*P_e_*	Typically 30–80% of *P*	Aggregate packing mode, CPT	Low *P_e_*: most pores are isolated, poor permeability; High *P_e_*: excellent drainage but risk of nutrient loss [106,109]
Pore size	2–8 mm	Aggregate gradation, target porosity	<2 mm: roots cannot penetrate, poor plant establishment; >8 mm: substrate instability, reduced strength [71,160]
Permeability coefficient	2–10 mm/s	*P*; *τ*; test method	<2 mm/s: inadequate drainage, runoff risk; >10 mm/s: potential nutrient washout, reduced water retention [22,113,161]
Compressive strength	5–25 MPa	*P*, paste quality, aggregate type, curing regime	<5 MPa: insufficient for structural stability; >30 MPa: often achieved at the expense of permeability (*P* < 15%) [71,162]

## Data Availability

No new data were created or analyzed in this study. Data sharing is not applicable to this article.
